# Liver Fibrosis and the Risks of Impaired Cognition and Dementia: Mechanisms, Evidence, and Clinical Implications

**DOI:** 10.3390/medsci14010044

**Published:** 2026-01-16

**Authors:** Mohamad Jamalinia, Ralf Weiskirchen, Amedeo Lonardo

**Affiliations:** 1 Gastroenterohepatology Research Center, Shiraz University of Medical Sciences, Shiraz 7193635899, Iran; mohamadjamalinia@gmail.com; 2 Institute of Molecular Pathobiochemistry, Experimental Gene Therapy and Clinical Chemistry (IFMPEGKC), RWTH University Hospital Aachen, D-52074 Aachen, Germany; rweiskirchen@ukaachen.de; 3 Department of Internal Medicine, Ospedale Civile di Baggiovara (-2023), Azienda Ospedaliero-Universitaria di Modena, Via Giardini 1135, 41125 Modena, Italy

**Keywords:** cognitive impairment, dementia, liver–brain axis, liver fibrosis, metabolic dysfunction, neuroinflammation, MASLD, gut–liver–brain axis, vascular dysfunction, non-invasive biomarkers

## Abstract

Liver fibrosis, the progressive accumulation of scar tissue resulting from chronic liver disease, is increasingly recognized as a multi-system condition, the effects of which extend beyond the liver, affecting brain health. Dementia, characterized by progressively impaired cognition sufficient to impede daily functioning, is a major global health issue with incompletely defined risk factors and pathogenic precursors. To examine the relationship between liver fibrosis and cognitive outcomes, we conducted a comprehensive PubMed literature search, and human studies published in English were included. Evidence is synthesized on the pathophysiology and clinical significance of liver fibrosis, types of dementia, and studies supporting the association between liver fibrosis and cognitive impairment. Meta-analytic data indicate that liver fibrosis is associated with an approximately 30% increased risk of incident dementia (pooled hazard ratio ~1.3), with progressively higher risks across more advanced fibrosis stages. Putative pathomechanisms, potentially modulated by age and sex, include chronic systemic and neuro-inflammation, insulin resistance, vascular dysfunction, and a perturbed intestinal microbiota–liver–brain axis. Non-invasive liver fibrosis diagnostics, advanced neuroimaging, and biomarkers represent key tools for assessing risk. In conclusion, liver fibrosis is a systemic condition that can affect brain health. Early detection, thorough risk assessment and interventions, such as lifestyle changes, metabolic therapies, and antifibrotic treatments, may help protect neural function. Key research gaps are identified, with suggestions for improving understanding of liver fibrosis’s connection to dementia or cognitive impairment.

## 1. Introduction

Liver fibrosis is the final consequence of repeated cycles of tissue damage and repair associated within chronic liver disease (CLD), regardless of etiology, and it is now being recognized as an important global health issue. Its incidence is increasing with the growing prevalence of metabolic dysfunction, older populations, and the burden of CLD related to alcohol and viral hepatitis [1]. Although historically described as a disease of exclusive hepatological significance, liver fibrosis exerts a considerable systemic impact on metabolic, cardiovascular, immune, and renal health which largely transcends the liver [2,3], and may also participate in central nervous system outcomes, including cognitive dysfunction and dementia [4].

Dementia, including Alzheimer’s disease and vascular dementia, is now one of the greatest public health challenges worldwide, affecting over 57 million individuals in 2019, with an incidence expected to rise to approximately 152 million by 2050 [5]. The pathophysiology of dementia is complex and multifactorial, including amyloid deposition, tau aggregation, neuroinflammation, microvascular dysfunction, and metabolic dysfunction [6]. Numerous underlying mechanisms are characterized by common biological pathways that become activated in cases of chronic liver injury, thereby providing a logical foundation for the existence of a liver–brain connecting axis [7]. For example, systemic inflammation, impaired ammonia metabolism, altered lipid and glucose pathways, and intestinal dysbiosis are not only well-characterized occurrences in advanced CLD but are also considered factors that contribute to neurodegeneration and cerebrovascular injury [8].

Only recently have studies specifically focused on the association between asymptomatic fibrosis detected by non-invasive methods in individuals without clinically overt cirrhosis (namely *subclinical liver fibrosis*) and long-term cognitive outcomes.

Several large-population studies now indicate that liver fibrosis, even including the precondition of non-cirrhotic livers, can be potentially associated with an increased risk of more rapidly progressive cognitive impairment and dementia [9,10]. This observation conceptualizes liver fibrosis as an important systemic determinant of brain aging rather than a condition of exclusive hepatological interest. Despite this accumulating evidence, significant research questions remain unanswered. The precise pathophysiological mechanisms linking liver fibrosis to cognitive decline are not fully understood. Further validation is needed to confirm the strength and consistency of epidemiological associations, and the potential of liver-related biomarkers to refine dementia risk stratification has not been fully explored. Additionally, the influences of patient heterogeneity, including sex, age, genetic background, and comorbidities, on the liver–brain relationship remain largely uncharacterized.

Given this complex background, the purpose of this review is to summarize the existing epidemiological, mechanistic, and clinical evidence to clarify the relationship between liver fibrosis and dementia. It also aims to identify relevant future directions that may potentially enhance early identification, risk stratification, and therapeutic approaches in both research and clinical practice.

## 2. Methods

We conducted a thorough literature search on PubMed to find studies that examine the connection between liver fibrosis and cognitive outcomes, including dementia and related neurodegenerative conditions. To ensure comprehensive and methodologically sound coverage, controlled vocabulary (MeSH terms) was utilized along with a wide range of titles and abstract keywords related to both liver fibrosis diagnostics and major dementia syndromes.

In the search for liver-related studies, we included the MeSH term “liver” along with MeSH headings on elasticity imaging techniques, biopsy, and fibrosis. Additionally, we used title and abstract keywords to capture non-invasive fibrosis methods and related terms such as *biopsy*, *fibrosis*, *cirrhosis*, *stiffness*, *elastography*, *acoustic radiation force impulse imaging*, *FibroScan*, *acoustography*, *vibroacoustography*, and *sonoelastography*.

For cognitive and neurodegenerative outcomes, the MeSH term “Dementia” was incorporated and the search was expanded to include a wide range of dementia-related conditions and synonyms used in the title and abstract fields. This encompassed common ley words such as *Alzheimer’s disease*, *vascular dementia*, and *frontotemporal lobar degeneration*, as well as terms related to less common neurodegenerative, prion-related, and hereditary dementias such as *CADASIL*, *Binswanger encephalopathy*, *Creutzfeldt–Jakob disease*, *progressive aphasia*, *tauopathies*, *neuronal ceroid lipofuscinosis*, *fatal familial insomnia*, *Huntington disease*, and the names of related disorders. These terms were chosen to ensure that all forms of cognitive impairment potentially linked to hepatic pathology were included.

The search was limited to human studies, publications in English, and adult populations, with no restrictions on study design. To cover historical evidence while including the most recent research, a publication date filter from 1 January 1900 to 30 November 2025 was applied. The research was structured using Boolean operators to maximize the retrieval of studies on the intersection of liver fibrosis and cognitive impairment, maintaining specificity using MeSH terms and targeted keywords such as those illustrated analytically in Table A1. A total of 176 PubMed records were identified and independently screened by the authors. The inclusion criteria were as follows: (1) original studies, including meta-analyses, conducted on humans and assessing the association between liver fibrosis and dementia; (2) liver fibrosis assessed via non-invasive biomarkers, imaging, or histology; and (3) outcomes including clinical dementia, mild cognitive impairment, neuroimaging correlates, and biomarker endpoints. Studies were excluded if they were narrative reviews, case reports, or conference abstracts. Any discrepancies were resolved through discussion among the authors.

For epidemiological studies included in this review, methodological quality and risk of bias were assessed using the Newcastle–Ottawa Scale (NOS). The NOS evaluates studies across three domains: the selection of study groups, comparability of groups, and ascertainment of exposure or outcomes. Studies achieving nine stars were considered to be at low risk of bias, those with seven to eight stars were considered to be at moderate risk of bias, and those with six stars or fewer were considered to be at high risk of bias. Other evidence was selected and discussed qualitatively based on relevance and biological plausibility.

## 3. Liver Fibrosis: Pathophysiology and Clinical Relevance

### 3.1. Stages

Liver fibrosis involves excessive buildup of extracellular matrix (ECM) proteins like collagen. While reversible, it may progress to cirrhosis, portal hypertension, and liver failure [11,12]. Fibrosis stages range from F0 (none) through to F4 (cirrhosis) [13], with significant fibrosis at F ≥ 2 and advanced fibrosis at F ≥ 3—important for comparing assessment methods [14].

### 3.2. Prevalence and Risk Factors

The prevalence rates of advanced fibrosis and cirrhosis in both sexes are shown in Table 1.

**Figure 1:**
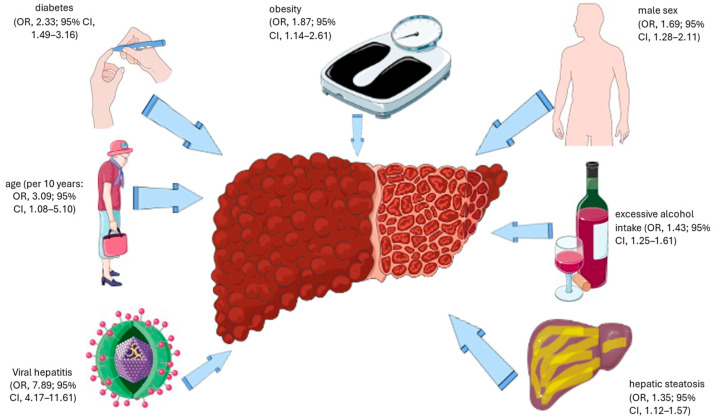
Principal risk factors for advanced liver fibrosis and cirrhosis. The original illustration was created using Servier Medical ART (SMART) and is licensed under the Creative Commons Attribution 4.0 International License (CC BY 4.0). The main risk factors for advanced liver fibrosis and cirrhosis are based on data from Zamani et al. [1]. Additionally, more rare causes of fibrosing liver disease include autoimmune, genetic, and drug-related etiologies of fibrosing chronic liver disease (CLD) [15]. In metabolic dysfunction-associated steatohepatitis (MASH), the independent predictors for significant and advanced fibrosis are waist circumference, metabolic syndrome, and alanine aminotransferase (ALT), age, platelets, Homeostatic Model Assessment (HOMA), diabetes, and total cholesterol, suggesting that systemic metabolic dysfunction is very closely associated with liver fibrosis [16]. Abbreviations: CI, confidence interval; OR, odds ratio.

Additionally, the prevalence of cirrhosis varies significantly among continents [1]. Figure 1 illustrates the principal risk factors for advanced liver fibrosis and cirrhosis [1]. Alarmingly, the prevalence of advanced fibrosis has increased over time from 2.0% before 2010 to 4.7% after 2016 [1], indicating an increasing burden at the level of the general population and clinically.

### 3.3. Pathomechanisms of Liver Fibrosis

Extra-hepatic and hepatic determinants drive liver fibrosis by activating hepatic stellate cells (HSCs) through their differentiation into myofibroblasts and the secretion of the ECM [17]. Visceral obesity contributes to hepatic fibrogenesis through lipotoxic and proinflammatory pathomechanisms involving genetics and epigenetics, an altered adipokine profile, oxidative stress, endoplasmic reticulum stress, and apoptosis [18,19]. The gut–liver axis significantly contributes to the histogenesis of cirrhosis through increased intestinal permeability, facilitated by gut dysbiosis, enabling bacteria to flow to the liver via the portal route [20], activating Kupffer cells via Toll-like receptor 4, and eventually leading to cytokine production, HSC activation, and liver fibrosis [21].

During fibrosis, immune cells and HSCs interact bi-directionally. Activated macrophages and neutrophils release signals that trigger HSC activation via inflammation [22]. “Hot” fibrosis exhibits immune cell infiltration and inflammation, while “cold fibrosis” is defined by minimal immune presence [23]. Gene variants like *PNPLA3* and cell stress responses play key roles in both the development and reversal of fibrosis [24,25]. HSCs experience stressors such as the unfolded protein response and oxidative stress, which prompt compensatory signaling [25]. Of concern is that liver fibrosis also increases the risk of hepatocellular carcinoma [26].

### 3.4. Liver Fibrosis and Extrahepatic Outcomes

Liver fibrosis dictates the natural course of liver-related outcomes and is a key modifier of systemic health and extra-hepatic events [27], including all-cause mortality [28], incident diabetes [29], major adverse cardiovascular events [30], extra-hepatic cancers [31,32,33] and chronic kidney disease [34]. In this evolving scenario, Jamalinia et al. have recently found that liver fibrosis is associated with a 32% long-term increased risk of dementia, irrespective of confounders, and that the severity of fibrosis worsens this risk [4].

## 4. Dementia: Overview

### 4.1. Definitions and Spectrum

Dementia is characterized by a progressive and persistent decline in cognitive abilities, including deficits in memory, executive function, language, visuospatial skills, and behavior. These impairments are significant enough to compromise daily independence, impacting essential activities such as self-care, monetary management, and household maintenance [35]. Importantly, dementia represents a chronic neurocognitive syndrome rather than a disease entity per se, encompassing a heterogeneous group of disorders with distinct yet often overlapping pathophysiological mechanisms [36].

Within the broader spectrum of cognitive disorders, dementia must be distinguished from delirium, an acute or subacute neuropsychiatric syndrome marked by fluctuating attention, altered levels of consciousness, and disorganized thinking [37]. Delirium is most commonly precipitated by reversible metabolic, infectious, or pharmacological insults and, unlike dementia, is potentially reversible with timely identification and correction of the underlying cause [37]. Although delirium and dementia may coexist, particularly in older individuals and patients with chronic systemic disease, their temporal course, diagnostic criteria, and management strategies differ fundamentally, underscoring the importance of accurate clinical differentiation [37]. Mild cognitive impairment (MCI) occupies an intermediate position along the cognitive continuum, defined by measurable cognitive decline that does not yet interfere with functional independence but confers an increased risk of progression to dementia [38].

**Figure 2:**
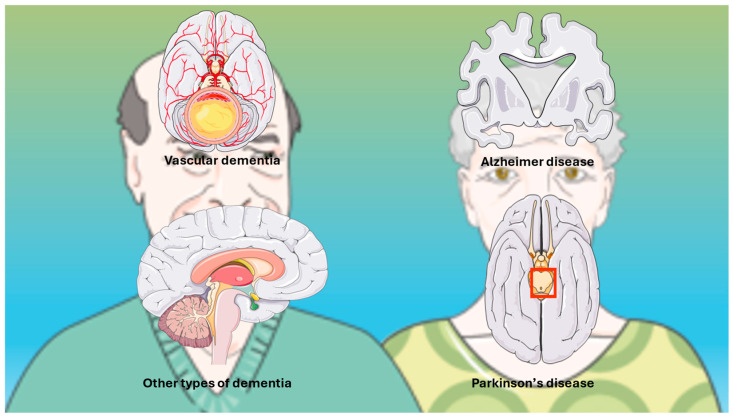
Categorization of dementia. Spectrum of the main subtypes of dementia based on references cited in the text. The original illustration was created using Servier Medical ART (SMART) and is licensed under the Creative Commons Attribution 4.0 International License (CC BY 4.0).

Among the dementia subtypes, Alzheimer’s disease (AD) is the most common subtype of dementia, comprising two-thirds of cases, and is defined by the presence of β-amyloid plaques and neurofibrillary tangles formed from hyperphosphorylated tau [39]. Vascular dementia (VaD), the second most common type of dementia, results from a microvascular cerebral pathology that encompasses small vessel disease, lacunar infarcts, and microbleeds [40]. Several other subtypes of dementia, including dementia with Lewy bodies [41], frontotemporal dementia [42], and mixed dementias, are particularly common in older individuals in whom processes of neurodegeneration and vascular pathology often coexist [43]. Parkinson’s disease (PD), the second most common neurodegenerative disorder after AD, is primarily a movement disorder. Many patients later develop PD dementia (PDD), usually years after PD diagnosis, particularly in those with late-onset, severe PD, mild cognitive impairment (MCI), depression, or sleep disorders [44,45,46]. Figure 2 schematically illustrates the principal dementia subtypes.

### 4.2. Pathophysiology and Risk Factors

Dementia arises from slowly progressive and sustained brain injury, driven by interacting neurodegenerative, inflammatory, and vascular processes [39]. In the case of AD, the accumulation of extracellular β-amyloid initiates a cascade of synaptic toxicity, oxidative stress, and microglial activation [47]. The abnormal phosphorylation of tau leads to impaired axonal transport, eventually inducing neuron dysfunction and death [48]. Neuroinflammation has emerged as a crucial player across the spectrum of dementia subtypes, as activated microglia and astrocytes secrete pro-inflammatory cytokines, enhancing neuron injury and impairing the clearance of toxic proteins [49,50]. Vascular pathology, including endothelial dysfunction, breakdown of the blood–brain barrier (BBB), cerebral hypoperfusion, and microvascular remodeling, plays an important role not only in VaD but also in accelerating cognitive impairment in AD [51,52]. Although each of these biological processes does not operate in isolation, they work together to alter neuronal health, synaptic connectivity, and brain network function, ultimately manifesting as clinical dementia. Importantly, while acute systemic disturbances may temporarily impair cognition (i.e., delirium), progression to dementia requires durable structural and molecular brain changes [53]. This underscores the distinction of dementia from reversible delirium [53].

There is also a wide range of modifiable and non-modifiable conditions that influence an individual’s progression to dementia. The strongest contributor remains age, with prevalence nearly doubling every 5 years after 65 [54]. Genetic risk, most commonly associated with the apolipoprotein E (APOE) ε4 allele genotype, significantly contributes to the risk of AD [55,56]. Cardio-metabolic risk factors and lifestyle habits, including atrial fibrillation, arterial hypertension, diabetes, dyslipidemia, obesity, and smoking, contribute to both AD and VaD, largely due to processes involving vascular injury, inflammation, and brain tissue hypoperfusion [57]. Other well-established risk factors include educational achievement, physical inactivity, social isolation, sensory deprivation, depression, and chronic systemic inflammation [58]. In addition to these established factors, metabolic dysfunction-associated steatotic liver disease (MASLD) may act as a systemic modifier of long-term brain vulnerability. This is due to its associated metabolic dysfunction, systemic inflammation, and gut–liver–brain interactions. This prompts research into the role of the liver in the pathogenesis of dementia [59,60].

## 5. Evidence Linking Liver Fibrosis and Dementia

### 5.1. Epidemiological Evidence

The epidemiological evidence linking liver fibrosis to dementia has significantly expanded over the past few years. Early insights came from cross-sectional studies showing that individuals with liver fibrosis perform poorly in cognitive domains relevant to dementia, such as executive function, attention, and memory. Additionally, they exhibit neuroimaging abnormalities like reduced cortical thickness, white matter microstructural changes, and markers of cerebral small-vessel disease [61,62,63,64] (Table 2). These initial observations laid the groundwork for subsequent longitudinal cohort analyses.

In recent years, an increasing number of population-based cohort studies have investigated whether liver fibrosis predicts incident dementia [4]. However, these studies have not produced entirely consistent results [4]. While some large cohorts have reported a significant positive association between fibrosis and later dementia [9,10,67,68], others have found non-significant relationships [69,70,71]. These discrepancies likely reflect differences in population characteristics, methods of diagnosing fibrosis (e.g., non-invasive scores vs. imaging-based assessment), the duration of follow-up, methods used to ascertain dementia, and residual confounding variables [4].

To address the uncertainties arising from these diverse findings, a recent meta-analysis was conducted, providing the most comprehensive and statistically powerful evaluation to date [4]. This analysis included eight cohort studies, involving approximately 1.1 million individuals, with around ~31,000 having liver fibrosis at the baseline. Over an average follow-up period of 14 years, approximately 30,000 incident dementia cases were recorded [4]. The combined results showed that liver fibrosis was linked to a roughly 30% increased risk of dementia (pooled Hazard Ratio [HR] 1.32; 95% Confidence Interval [CI] 1.08–1.61), independent of demographic, socioeconomic, anthropometric, and cardiometabolic factors [4].

Importantly, the risk of dementia increased progressively in parallel with the severity of liver fibrosis [4]. Pooled estimates revealed rising hazard ratios across fibrosis stages: HR 1.06 (95% CI 0.67–1.68) for ≥F2, HR 1.32 (95% CI 1.06–1.64) for ≥F3, and HR 1.69 (95% CI 1.01–2.83) for F4 [4]. Sensitivity analyses, limited to studies with maximum covariate adjustments, confirmed the strength and independence of this association (*n* = 5 studies; pooled HR 1.29, 95% CI 1.07–1.56) [4]. Overall, these findings establish liver fibrosis as an independent and clinically significant predictor of long-term dementia risk, providing the most compelling epidemiological evidence to date for this emerging liver–brain pathogenic axis [4].

### 5.2. Mechanistic Insights

Mechanistic links between liver fibrosis and cognitive impairment are established through validated animal studies, human biomarker and neuroimaging data, and hypotheses based on epidemiological and pathophysiological evidence.

**Figure 3:**
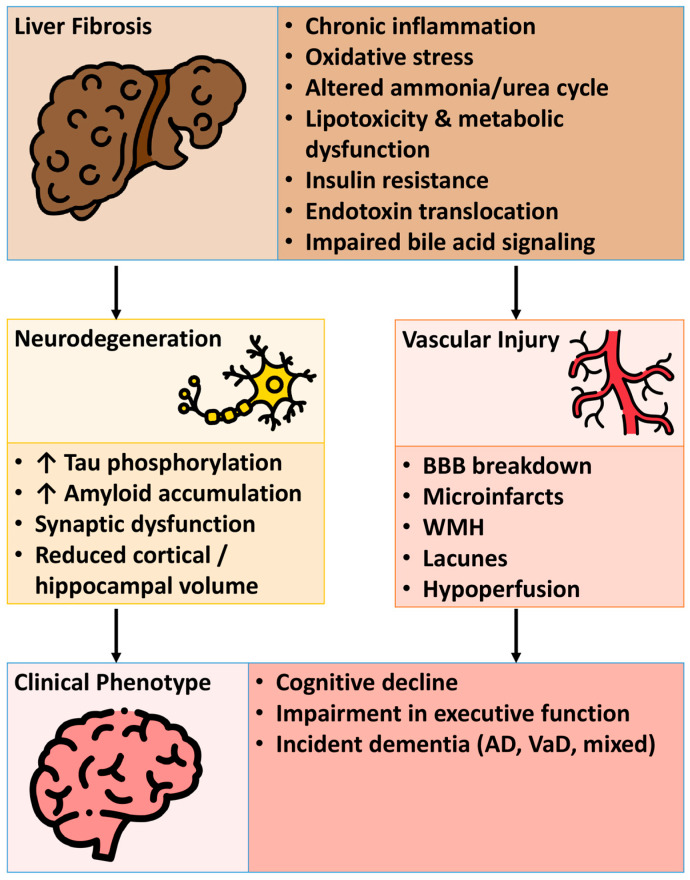
Putative mechanistic pathways linking liver fibrosis with the risk of dementia. This figure illustrates the complex biological pathways through which liver fibrosis may contribute to accelerated brain aging and an increased risk of dementia. The pathways integrate metabolic, vascular, inflammatory, and neurotoxic mechanisms described throughout the manuscript, highlighting how hepatic dysfunction can influence cerebral structure, function, and neurodegenerative processes. Abbreviations: BBB, blood–brain barrier; WMH, white matter hyperintensity; AD, Alzheimer’s disease; VD, vascular dementia. The icons used in this figure were sourced from flaticon.com (Accessed Date: 8 January 2026).

Liver fibrosis contributes to dementia through interconnected pathways involving neuroinflammation, insulin resistance, vascular dysfunction, oxidative stress, and a perturbed gut–liver–brain axis. These diverse pathomechanisms are schematically illustrated in Figure 3.

#### 5.2.1. Liver–Brain Axis: Neuroinflammation, Insulin Resistance, and Vascular Dysfunction

Experimental studies in animal models and translational human research suggest that fibrotic CLD triggers a chronic inflammatory environment. In this environment activated Kupffer cells and HSCs release tumor necrosis factor-α (TNF-α), interleukin-6 (IL-6), and C-reactive protein (CRP) [72,73]. These substances can easily cross the BBB and activate microglia, shifting the balance towards a pro-inflammatory state. Microglial activation has been demonstrated in experimental systems to accelerate β-amyloid aggregation and tau hyperphosphorylation, providing a mechanistic explanation consistent with Alzheimer’s disease pathology [74]. Furthermore, systemic insulin resistance, a characteristic of MASLD, impairs insulin receptor signaling in neurons, reduces brain glucose uptake, and worsens mitochondrial dysfunction. Cerebrovascular damage acts as a third contributing factor [75]. Specifically, portal hypertension and endotoxemia increase levels of vasoconstrictors like endothelin-1, while reducing nitric oxide availability [72]. This results in endothelial dysfunction and small vessel disease, leading to clinical manifestations such as white matter hyperintensities and lacunar infarcts [73]. These combined processes create an unfavorable cerebral environment that heightens the risks of neurodegeneration, explaining why more severe stages of fibrosis are linked with a higher risk of dementia in epidemiological studies [67,76]. While these pathways are supported by experimental and translational data, their relative contribution to dementia risk in humans with liver fibrosis remains partially inferential and hypothesis-generating.

#### 5.2.2. Metabolic Dysregulation and Oxidative Stress

Beyond its inflammatory effects, chronic liver injury is often accompanied by profound alterations in systemic metabolic function that vary based on the etiology of CLD. For example, MASLD, the prototypic hepatic manifestation of metabolic dysfunction, disrupts lipid handling, resulting in atherogenic dyslipidemia characterized by elevated very-low-density lipoprotein particles (VLDL), low-HDL cholesterol, and oxidized low-density lipoprotein (LDL) [77,78]. These lipoproteins can accumulate in cerebral vessels and brain parenchyma, further amplifying oxidative stress [79]. Meanwhile, impaired hepatic β-oxidation leads to the spill-over of free fatty acids, which activate NADPH oxidase and produce reactive oxygen species (ROS) both in the liver and peripherally [80,81]. Much of the evidence linking oxidative stress, dyslipidemia, and neuronal injury comes from experimental and mechanistic studies. Conversely, data in humans remain largely associative rather than causative. ROS rapidly deplete antioxidants such as glutathione and superoxide dismutase, leaving neurons susceptible to peroxidative injury [82,83]. Compounding this vulnerability, fibrotic CLDs exhibit impaired capacity to synthesize ceruloplasmin and transferrin, resulting in dysregulated iron and copper metabolism. These elements catalyze Fenton reactions and exacerbate oxidative DNA damage in neural tissue [84]. Collectively, these metabolic derangements create a systemic pro-oxidant state that synergizes with neuroinflammation to accelerate neuronal apoptosis and synaptic loss [74]. Therefore, these metabolic mechanisms should be viewed as biologically plausible contributors rather than definitively proven causal pathways in human dementia.

#### 5.2.3. Gut–Liver–Brain Axis: Intestinal Microbiota, Endotoxins, and Ammonia

The intestinal microbiota serves as an increasingly recognized connection between hepatic and cerebral pathology [85]. During liver fibrosis, intestinal permeability increases due to portal hypertension and mucosal congestion, allowing bacterial products like lipopolysaccharide (LPS) and peptidoglycan to invade the portal and systemic circulation [85]. LPS activates Toll-like receptor-4 on Kupffer cells, intensifying hepatic inflammation while simultaneously weakening the BBB through the cytokine-mediated disruption of tight junctions [86]. Gut dysbiosis also leads to the overgrowth of urease-producing bacteria that release ammonia. Although blood levels of ammonia do not necessarily predict clinical symptoms, chronic low-grade exposure can interfere with astrocytic glutamine synthetase and disrupt neurotransmission [87]. Additionally, microbial metabolites like trimethylamine-N-oxide (TMAO) can increase platelet reactivity and vascular inflammation, connecting gut changes to both hepatic and cerebrovascular damage [88]. The decreased production of short-chain fatty acids such as butyrate eliminates an essential anti-inflammatory and neurotrophic signal, further shifting the balance towards neurodegeneration [89,90]. Therefore, the gut–liver–brain axis provides a reliable biological framework supported by experimental evidence. However, prospective studies in humans are needed to confirm causality.

### 5.3. Sex and Age Differences

Sex and age are key factors in shaping the relationship between liver fibrosis and dementia, influencing both conditions through biological, hormonal, and lifestyle mechanisms. Sex plays a role in the development of common types of CLD, such as MASLD, while also affecting the risk of dementia. Meanwhile, age is a significant risk factor for both conditions, interacting with hormonal and genetic factors to influence the progression of dementia.

Sex and gender impact liver fibrosis through various factors like genetics, hormones, immune response, metabolism, and lifestyle factors, including alcohol consumption, diet, physical activity, and hormone therapy [91]. Women have a higher risk and prevalence of AD compared to men, with notable differences in disease progression and response to treatment [92].

Age can accelerate liver disease progression and worsen cognitive issues by reducing liver volume and blood flow, impairing detoxification, and affecting metabolic function. These factors increase the risk of conditions like hepatic encephalopathy and neurodegenerative diseases [76,93]. Additionally, age-related hormonal changes, such as declines in androgen in men, can impact both liver and brain health simultaneously [94,95,96,97]. Aging also increases susceptibility to acute liver injury, fibrosis, and poor outcomes in CLD due to various factors [98]. Similarly, aging raised the risk of neurodegenerative diseases like AD and Parkinson’s due to genomic instability, telomere shortening, epigenetic changes, proteostasis loss, mitochondrial dysfunction, cellular senescence, disrupted nutrient sensing, stem cell depletion, and altered cell communication [99]. Liver fibrosis may affect dementia risk differently based on age and sex [4]. Hormones like estrogen and androgen play a role in the risk of liver disease and dementia as individuals age [100,101]. In women, hormonal changes after menopause largely explain the increased risk of dementia in older age [102].

## 6. Diagnostic Considerations

### 6.1. Fibrosis Assessment

**Figure 4:**
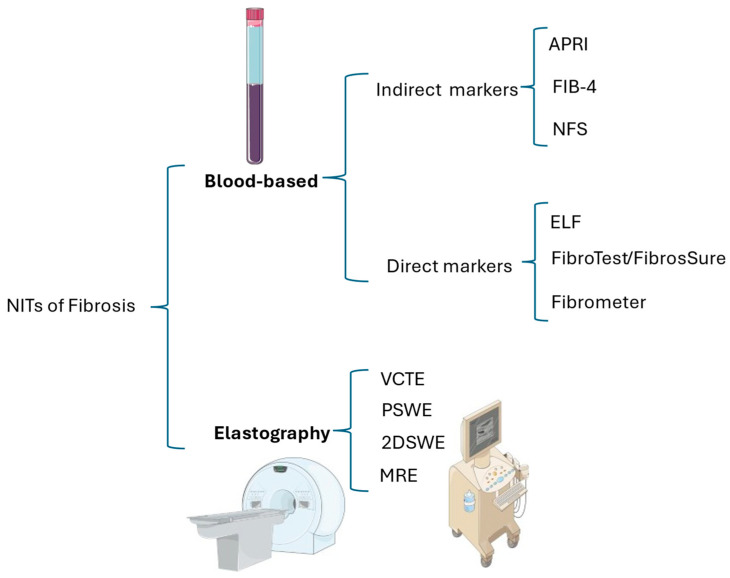
Schematic illustration of the most frequently used non-invasive tests for liver fibrosis. The original illustration was created using Servier Medical ART (SMART) and licensed under the Creative Commons Attribution 4.0 International License (CC BY 4.0). Abbreviations: APRI, aminotransferase–platelet ratio index; ELF, enhanced liver fibrosis score; FIB-4, fibrosis-4 index; MRE, magnetic resonance elastography; NFS, NAFLD fibrosis score; PSWE, point shear wave elastography; 2DSWE, two-dimensional shear wave elastography; VCTE, vibration-controlled transient elastography.

Historically, liver fibrosis was identified solely on histological grounds, but now it is increasingly detected non-invasively in medical research and clinical practice. Currently, fibrosis assessment can be divided into two categories: biopsy-proven fibrosis and surrogate-based fibrosis. The latter is diagnosed using blood-based biomarker tests and liver stiffness measurements assisted by imaging techniques like elastography. The risks and limitations of liver biopsy have led to research on and validation of noninvasive tests (NITs) that can detect fibrosis and cirrhosis at asymptomatic stages [103,104,105]. The recent Food and Drug Administration (FDA) initiative to consider proposals for using NITs instead of liver histology as reference standard endpoints (RLSEs) provides strong motivation for the adoption of NITs in drug development for metabolic dysfunction-associated steatohepatitis [106]. Figure 4 categorizes NITs for fibrosis as either blood-based or based on imaging techniques.

#### 6.1.1. Blood-Based Non-Invasive Tests

The most thoroughly validated blood-based noninvasive tests include APRI, FIB-4, and ELF [105]. The practical benefits of these methods include applicability to over 95% of patients, reliable reproducibility, extensive accessibility, and, specifically for APRI and FIB-4, cost-effectiveness, due to their calculation from routine blood tests [105]. Nevertheless, increasing reliance on these surrogate markers is associated with a non-negligible risk of misclassification, particularly in specific patient populations, such as older individuals and those with type 2 diabetes (T2D) [107]. These scores can be affected by confounding variables, such as age with FIB-4 and both age and extrahepatic fibroinflammatory changes with ELF [105,108]. A decreased accuracy of NIT is also observed among subjects with T2D because of specific characteristics of this patient population, and owing to the effects of T2D itself on some NIT biomarkers of fibrosis [109]. Accordingly, interpretations of blood-based NITs in older patients and those with T2D should be made cautiously and preferably integrated into sequential diagnostic algorithms incorporating imaging-based elastography or histological confirmation [107]. Furthermore, the ELF test, being commercially available, is comparatively costly [105]. Table 3 summarizes the principal features of the blood-based indirect NITs.

#### 6.1.2. Elastometry

Liver stiffness can be assessed through various ultrasound-based elastography techniques or magnetic resonance elastography (MRE) [114]. Liver stiffness measurements are reported in kilopascals (kPa), with values below 5 kPa considered within the normal range. These measurements are more prone to producing false positives than false negatives [105].

The practical benefits of vibration-controlled transient elastography (VCTE) include its point-of-care accessibility, straightforward learning curve, and consistent reliability, exceeding 95% when an extra-large probe is applied in patients without morbid obesity [115]. Nonetheless, it is essential to thoroughly account for potential confounding factors to prevent an inaccurate assessment of fibrosis [105].

Other ultrasound elastography methods, such as point shear wave and two-dimensional shear wave elastography, have diagnostic accuracy comparable to that of VCTE [116]. However, differences between platforms, variable cutoffs, and limited validation restrict their widespread adoption [105].

#### 6.1.3. Sequential Non-Invasive Assessment of Liver Fibrosis

A two-step protocol is typically applied to triage individuals displaying “red flags” for CLD. The protocol includes initial testing (APRI for individuals with viral hepatitis and FIB-4 for others) followed by secondary testing based on history, laboratory liver tests, and ultrasonography scanning [105]. The FIB-4 index uses low and high cutoffs to rule out or confirm advanced fibrosis, with values in between considered indeterminate [105]. Sequential combinations of markers with lower thresholds to exclude advanced fibrosis and higher thresholds to confirm cirrhosis can decrease the need for liver biopsies [117].

Patients with indeterminate or high FIB-4 scores should undergo further noninvasive testing (VCTE/MRE) or biopsy. VCTE is the most validated elastography method for detecting advanced fibrosis [105,118]. A liver stiffness value under 15 kPa with platelet counts above 150,000/mm^3^ excludes significant portal hypertension, while a stiffness value over 25 kPa confirms portal hypertension in cirrhotic patients [105].

## 7. Cognitive Assessment and Biomarkers

Accurate outcome classification is crucial when examining liver–brain associations, as cognitive impairment can vary in temporal profiles, biological substrates, and prognostic implications. Misclassification, especially when chronic neurodegenerative outcomes are confused with acute cognitive syndromes, can significantly bias interpretation and appropriate management [119]. Therefore, cognitive assessments and biomarkers need to be interpreted within their intended diagnostic framework.

### 7.1. Mini-Mental State Examination and Montreal Cognitive Assessment

The Mini-Mental State Examination (MMSE) and the Montreal Cognitive Assessment (MoCA) evaluate memory, orientation, and language abilities and are commonly used to identify mild cognitive impairment (MCI) and dementia [120]. Both the MMSE and MoCA are suitable cognitive assessment tools for monitoring changes in cognition [121]. However, these instruments are not intended to characterize acute cognitive syndromes that occur during systemic illness [122,123]. Therefore, reduced MMSE or MoCA performance in medically unstable settings should be interpreted with caution and considered within the broader clinical context [122,123]. Dedicated delirium-focused assessments and structured clinical evaluations are necessary when acute cognitive changes are suspected [123]. Follow-up assessments of any altered cognitive states over time may help differentiate transient brain failure from irreversible or progressive dementia in individual cases.

A study of 803 German-speaking Memory Clinic outpatients discovered that MoCA scores were consistently lower than MMSE scores. The study also introduced a simple conversion table for comparing cognitive test results in patients with neurocognitive disorders [120]. This data supports the preferential use of MoCA in identifying prodromal neurodegenerative change rather than short-lived cognitive disturbances.

### 7.2. Diagnostic Modalities and Biomarkers: Chronic Neurodegeneration Versus Transient Cognitive Dysfunction

Beyond screening instruments, the etiological attribution of cognitive impairment relies on the integration of neurological examinations, neuroimaging techniques such as CT and MRI scans, and, in certain cases, cerebrospinal fluid or blood tests to exclude alternative diagnoses and determine potential underlying causes [124]. These modalities are particularly informative for distinguishing slowly evolving neurodegenerative processes from cognitive impairment driven by acute metabolic, infectious–inflammatory, or toxic insults.

Recent meta-analyses emphasize the growing relevance of blood- and CSF-based biomarkers in characterizing MCI and early dementia (Table 4). Markers such as neurofilament light chain (NfL), glial fibrillary acidic protein (GFAP), amyloid-β species, and tau reflect axonal injury, astroglial activation, and protein aggregation—pathophysiological processes that develop over prolonged periods and align with chronic neurodegeneration rather than acute cognitive syndromes [125,126].

By contrast, several inflammatory and metabolic biomarkers lack outcome specificity, as they may be elevated during both chronic neurodegenerative states and acute systemic stress [127,128,129]. Interpretation, therefore, requires longitudinal assessment and corroboration with neuroimaging and cognitive follow-up to avoid attributing transient cognitive impairment to irreversible disease processes. Framing biomarker evidence within this temporal and mechanistic context is essential for accurate outcome classification.

**Table 4:** Meta-analytic evidence supporting the use of biomarkers for diagnosing dementia.

Author, Year [Ref]	Method	Findings	Conclusion
Gaur, 2023 [130]	Meta-analysis of 10 studies	In CSF, concentrations of NfL (SMD = 0.69 [0.56, 0.83]), GFAP (SMD = 0.41 [0.07, 0.75]), and HFABP (SMD = 0.57 [0.26, 0.89]) were elevated in individuals with MCI. In blood, increased concentrations of T-tau (SMD = 0.19 [0.09, 0.29]), NfL (SMD = 0.41 [0.32, 0.49]), and GFAP (SMD = 0.39 [0.23, 0.55]) were found in MCI.	Levels of NfL and GFAP can be measured in both CSF and blood. Monitoring these biomarkers may provide valuable information about neurodegeneration in individuals with MCI.
Ma, 2024 [131]	Meta-analysis of 63 studies	The following biomarkers were significantly higher in patients with PSCI compared to the non-PSCI group: Hcy (*p* < 0.00001), CRP (*p* = 0.0008), UA (*p* = 0.02), IL-6 (*p* = 0.005), Cys-C (*p* = 0.0001), creatinine (*p* < 0.00001) and TNF-α (*p* = 0.02).	Integrating neuroimaging and neuropsychological assessments with blood biomarker levels is crucial for evaluating the risk of PSCI.
Chen, 2024 [132]	Meta-analysis of 13 studies	A notable elevation in MI concentration was found, along with reductions in Glu, Glx, and NAA/Cr ratios in DCI.	These biomarkers are highly sensitive metabolic indicators for assessing the progression of DCI.
Huang, 2025 [133]	Meta-analysis of 30 studies	Peripheral Aβ42 levels, the Aβ42/Aβ40 ratio, NfL, and S100B showed significant differences between VCI and non-VCI groups.	Peripheral Aβ42, the Aβ42/Aβ40 ratio, NfL, and S100B are potential blood biomarkers for VCI.

Abbreviations: Aβ40, amyloid beta 40; Aβ42, amyloid beta 42; CRP, C-reactive protein; CSF, cerebrospinal fluid; Cys-C, cystatin C; DCI, diabetic cognitive impairment; GFAP, glial fibrillary acidic protein; Glu, glutamate; Glx, composite of glutamate and glutamine; GFAP, glial fibrillary acidic protein; Hcy, homocysteine; HFABP, heart-type fatty acid-binding protein; IL-6, interleukin-6; TNF-α, tumor necrosis factor-α; MCI, mild cognitive impairment; MI, myo-inositol; NAA/Cr, N-acetylaspartate/creatine; NfL, neurofilament light chain; PSCI, post-stroke cognitive impairment; S100B, S100 calcium-binding protein B; SMDs, standardized mean differences; TNF-α, tumor necrosis factor-alpha; UA, uric acid; VCI, vascular cognitive impairment.

### 7.3. Risk Prediction Models

Dementia risk prediction models estimate long-term susceptibility to neurodegenerative disease by integrating demographic, vascular, metabolic, and lifestyle factors. These tools are calibrated to predict future dementia risk and are not intended to capture acute or reversible cognitive outcomes. Validated dementia risk algorithms that assist clinicians in stratifying patients are listed in Table 5. It is important to note that none of these widely used models currently include any liver-related biomarkers or fibrosis measurements. However, a recent meta-analysis has shown that liver fibrosis is linked to cognitive impairment independently of the variables already used in these scores [4]. This suggests that incorporating liver fibrosis parameters, such as VCTE-based stiffness values, into future risk models could lead to the early identification of individuals at a heightened risk of dementia. Nevertheless, the addition of variables does not always improve model discrimination or calibration. Therefore, future research should assess whether liver fibrosis markers add prognostic value for dementia beyond known predictors, and if their effects are specific to chronic neurodegenerative outcomes rather than acute illness-related cognitive disturbances.

## 8. Therapeutic and Preventive Implications

### 8.1. Liver-Directed Interventions: Lifestyle, Pharmacological, and Bariatric Approaches

Lifestyle modifications remain the cornerstone of antifibrotic therapy [141]. Sustained 7–10% weight loss, achieved through Mediterranean-style diets and structured aerobic resistance exercise regimens, can induce histologic fibrosis regression in up to one-third of patients [142]. Additionally, this approach improves executive function and memory scores on Montreal Cognitive Assessment (MoCA) testing [143]. Pharmacologically, glucagon-like peptide-1 receptor agonists (GLP-1 RAs) have become a primary focus. For example, semaglutide has been shown to reduce the histological activity of steatohepatitis, lower FIB-4 scores, and demonstrate preliminary slowing of cognitive impairment in T2D cohorts [144]. Farnesoid-X-receptor agonists like obeticholic acid and fibroblast growth factor-19 analogs (e.g., aldafermin) are currently undergoing phase III evaluation with secondary neurocognitive endpoints [145,146,147]. Bariatric surgery is the most effective and durable option for patients with morbid obesity and advanced fibrosis. Meta-analyses have indicated that Roux-en-Y gastric bypass and sleeve gastrectomy can reduce excess weight, total weight, cirrhosis progression, and decrease incident dementia by roughly 25–30% over a decade [148,149]. These effects are mediated by improvements in insulin sensitivity, reduced systemic inflammation, and favorable alterations in gut microbiota. It is crucial to monitor post-operative micronutrient levels to prevent B-vitamin deficiencies that could potentially negate cognitive gains [150,151].

### 8.2. Neuroprotective Potential of Liver-Focused Therapies

Mounting evidence suggests that therapies aimed at the liver can have direct and indirect neuroprotective effects. Resolving hepatic inflammation reduces systemic cytokine burden, decreases microglial activation, and preserves synaptic integrity [75]. Improved insulin sensitivity also boosts cerebral glucose uptake, supporting neuronal energy metabolism [152]. In animal models, GLP-1 RAs have been shown to cross the BBB, increase cyclic-AMP response element binding protein (CREB) levels, and stimulate hippocampal neurogenesis [153]. Antioxidant agents like *N*-acetyl-cysteine, S-adenosyl-L-methionine, and manganese porphyrins are being investigated for their antifibrotic properties, as they can simultaneously reduce ROS levels in both the liver and brain [154,155]. Modulating the intestinal microbiota through high-fiber diets, prebiotics or, potentially, fecal microbiota transplantation can increase short-chain fatty acid production, which binds to G-protein-coupled receptors on microglia and promotes an anti-inflammatory response [156,157]. Taken together, these findings suggest that targeting hepatic fibrosis could potentially delay the onset or slow the progression of both AD type and vascular dementias. This hypothesis is currently being tested in umbrella trials that assess hepatic, metabolic and cognitive outcomes [158].

### 8.3. Multidisciplinary Care: Integrating Hepatology and Cognitive Medicine

Given the multifactorial nature of the fibrosis–dementia nexus, siloed care models are insufficient. An integrated pathway should begin with dual screening: non-invasive fibrosis assessment (using transient elastography or serum panels such as FIB-4) alongside cognitive testing (such as MoCA or digital neuropsychological batteries) in primary-care or diabetology settings [9,159]. Patients with either abnormality should be referred to combined hepatology–neurology clinics, where liver ultrasonography, MRE, and brain MRI with diffusion tensor sequences can be ordered during a single visit.

Multidisciplinary teams, comprising hepatologists, neurologists, endocrinologists, dietitians, and clinical psychologists, should collaborate to develop personalized care plans that address lifestyle factors (such as healthy diets, increased physical activity, and reduced sedentary time), pharmacotherapy, and psychosocial support [160,161]. Electronic health record dashboards should support real-time monitoring of liver stiffness, metabolic parameters, and cognitive scores, allowing for prompt adjustments to therapy. This integrated approach not only enhances the patient experience but also has the potential to identify treatable factors (such as obstructive sleep apnea or vitamin D deficiency) that impact both liver and cognitive function [162,163]. Additionally, public health campaigns focused on viral hepatitis vaccination, alcohol harm reduction, and metabolic syndrome screening could result in decreased rates of cirrhosis and dementia at a population-level, underpinning the interconnected nature of liver and brain health [164,165,166,167].

## 9. Gaps in Knowledge and Future Directions

Although evidence links liver fibrosis to increased dementia risk, key information is still missing to fully understand these connections and apply them clinically.

A major challenge is that the majority of existing epidemiological studies are based on indirect and non-invasive assessments of liver fibrosis, e.g., FIB-4 or the NAFLD fibrosis score (NFS), which pose the significant risk of misclassification and fail to capture changes in the temporal course of CLD, thus risking an underrepresentation of the real relationship between liver fibrosis and cognitive impairment [4,107].

Establishing the temporal course of liver fibrosis development through longitudinal cohort studies with repeated, accurate and easily accessible assessments of fibrosis (with an emphasis on elastography) is necessary to characterize the relationship between liver fibrosis progression and cognitive loss [168].

Currently, the precise pathomechanisms by which liver fibrosis contributes to the development of dementia remain incompletely characterized.

Only a limited number of studies have examined the contribution of inflammatory, vascular, and metabolic dysregulation, and gut–liver–brain pathways [169], although many potential mechanisms may be at play. We believe that multi-omic and imaging approaches may help determine the causal mediators of the relationship between liver disease and cognitive impairment, as well as the common pathways connecting them and identifying early biomarkers indicating susceptibility to neurodegeneration.

Another important unanswered question is related to patient population heterogeneity. The distribution of variables associated with sex, age, hormonal status, and genetic background in the population, which will affect liver disease development over time and the risk of developing dementia, is infrequently taken into account during the analyses conducted or the study designs within the existing literature [4,170]. Additionally, the impact of various causes of CLD [MASLD, alcohol-related Liver Disease (ALD), metabolic dysfunction, and ALD (MetALD)], viral hepatitis, and more rare etiologies [171,172] is not well defined in existing studies, and stratified analyses based on the etiology of liver disease will likely uncover different risk characteristics and distinct disease mechanisms.

Our review was limited to studies published in English and indexed in PubMed, which may have excluded relevant evidence from other languages or databases. Additionally, thematic overlaps with other pathologies, terminological inconsistencies, heterogeneous study designs, variable fibrosis assessment methods, and cognitive outcomes further complicate the interpretation of findings. Despite these limitations, we aimed to provide a comprehensive and updated overview highlighting consistent patterns and knowledge gaps to guide future research.

There are also more areas of therapeutic implications in need of investigation. It is unclear if lifestyle modifications, newer antifibrotic therapies, metabolic agents and/or bariatric surgery used to improve the liver’s fibrotic state will reduce cognitive impairment. Therefore, studies with cognitive endpoints/cognition-related biomarkers should ascertain whether liver-based therapies would provide neuroprotective effects.

Another emerging area of data analytics is the utilization of machine learning and big data methodologies. By utilizing large-dataset approaches, which include electronic medical records with neuroimaging, as well as genetic and longitudinal cognitive data, we can improve predictive models of dementia risk in individuals with liver disease [173,174,175]. Nevertheless, thorough validation and standardization of these models is required prior to their implementation in routine clinical practice.

Bridging knowledge gaps in our understanding of the link between fibrosing CLD and progressive cognitive impairment or dementia is essential for clarifying causality, improving risk assessment, and guiding prevention and treatment strategies for both liver and brain health.

## 10. Conclusions

Liver fibrosis is increasingly recognized as a systemic disorder with effects beyond the liver, potentially impacting brain health. Epidemiological and biological evidence supports an association between liver fibrosis, including non-cirrhotic stages, and increased risk of cognitive impairment and dementia. Shared mechanisms such as chronic inflammation, metabolic dysfunction, vascular and oxidative damage, and gut–liver–brain axis disruption likely contribute to this relationship. Early detection of liver fibrosis, comprehensive risk assessment, and non-invasive testing may help guide preventive strategies. Emerging therapies for MASLD and fibrosis, along with lifestyle and metabolic interventions, hold potential for neuroprotection. However, knowledge gaps remain regarding the timing, duration, and type of liver injury, as well as major risk modifiers of dementia such as sex, age, and genetic factors. Further studies, including Mendelian randomization analysis and interventional trials, are needed to clarify causal mechanisms while also evaluating the effects of improved liver health on the prevention or retardation of cognitive decline [176].

## Figures and Tables

**Table 1:** Meta-analytic estimates of advanced fibrosis and cirrhosis in men and women [1].

	Global Prevalence of Advanced Fibrosis	Global Prevalence of Cirrhosis
**Overall**	3.3% (95% CI, 2.4–4.2)	1.3% (95% CI, 0.9–1.7)
**In men**	3.5% (95% CI, 2.6–4.5)	2.5% (95% CI, 1.0–4.0)
**In women**	2.2% (95% CI, 1.3–3.1)	0.9% (95% CI, 0.0–1.8)

Abbreviations: CI, confidence interval.

**Table 2:** Summary of key studies linking liver fibrosis and metabolic liver disease to brain structure, cognition, and Alzheimer’s disease pathology.

Author, [Ref]	Method	Findings	Outcome Classification	Risk of Bias	Comment
Jamalinia et al. [4]	Meta-analysis of eight diverse cohorts diagnosing liver fibrosis non-invasively or via histology. Primary outcome: new-onset dementia	Eight longitudinal cohorts, including 1,115,759 middle-aged individuals (31,129 with liver fibrosis at baseline), identified 29,923 new dementia cases over a mean follow-up of 14 years. Liver fibrosis exhibited a 32% increased risk of developing all-cause dementia (pooled HR: 1.32; 95% CI: 1.08–1.61; I^2^ = 76.06%). Dementia risk increased with fibrosis severity: HR 1.06 in ≥F2, HR 1.32 in ≥F3, and HR 1.69 in F4. The magnitude of risk tends to be higher in women (HR 1.94) than in men (HR 1.18).	Clinical Dementia	Moderate to low	Provides the strongest epidemiological evidence to date linking liver fibrosis to long-term risk of clinical dementia.
Vataja et al. [65]	Finnish nationwide Health 2000/2011 Surveys. Cognitive tests: verbal fluency, word-list learning (WLL), delayed recall, and reaction times.	Cross-sectionally (5139 participants), there were no significant associations; however, longitudinally (3143 participants), baseline MASLD (FLI > 60) predicted poorer WLL and decline over time (*p* < 0.04).	MCI	Low	Provides population-level evidence that MASLD predicts decline in working memory.
Parikh et al. [66]	UK Biobank study. Liver fibrosis assessed via FIB-4. Primary cognitive outcome: Digit Symbol Substitution Test (DSST); secondary: executive function, processing speed, memory. Imaging: hippocampal, total brain, WMH volumes	105,313 participants with cognitive tests; 41,982 with MRI. Liver fibrosis was associated with worse DSST and executive function, not memory. Lower hippocampal and total brain volumes, with no clear WMH association.	MCI, Neuroimaging	Moderate	Demonstrates executive function and structural brain changes associated with liver fibrosis.
Weinstein et al. [62]	A cross-sectional meta-analysis was conducted on 5660 individuals with MASLD and 3022 individuals with fibrosis, who were free of dementia and stroke, from the FHS, RS, and SHIP cohorts. MASLD was assessed using abdominal imaging, while fibrosis was assessed using FibroScan.	MASLD is associated with smaller total brain volume (β = −3.5, 95% CI −5.4 to −1.7), gray matter volume (β = −1.9, 95% CI −3.4 to −0.3), and cortical gray matter volume (β = −1.9, 95% CI −3.7 to −0.01). Fibrosis (liver stiffness ≥ 8.2 kPa) is linked to smaller total brain volume (β = −7.3, 95% CI −11.1 to −3.5). There is low heterogeneity.	Neuroimaging	Low	Suggests that MASLD and fibrosis may play a role in brain aging.
Fan et al. [63]	A cross-sectional study was conducted on 29,195 UK Biobank participants aged 45–82 who underwent T1, T2 FLAIR, and DTI MRI scans. MASLD was defined as MRI-PDFF ≥5% plus ≥1 cardiometabolic criterion.	MASLD is associated with smaller total/subcortical gray matter (*p* < 0.05), reduced AD-signature cortical thickness (β = −0.04), higher WMH (β = 0.12), increased FA (β = 0.05), and reduced MD (β = −0.04).	Neuroimaging	Low	Confirms MASLD affects gray and white matter integrity, emphasizing structural brain correlates.
Weinstein et al. [64]	Participants from the Framingham Offspring and Third Generation cohorts underwent amyloid (11C-PiB) and tau (18F-Flortaucipir) PET scans, as well as abdominal CT scans, or had FIB-4 data.	FIB-4 is associated with increased rhinal tau levels (β = 1.03 ± 0.33, *p* = 0.002). In MASLD participants, higher FIB-4 levels are correlated with increased tau in regions such as the inferior temporal (β = 2.01 ± 0.47), parahippocampal (β = 1.60 ± 0.53), entorhinal (β = 1.59 ± 0.47), and rhinal cortex (β = 1.60 ± 0.42), as well as increased overall amyloid-β (β = 1.93 ± 0.47).	Biomarker-Based Endpoints	Moderate	Demonstrates that liver fibrosis may drive early Alzheimer’s pathology, linking liver disease and neurodegeneration.

Note: Throughout this table, the term metabolic dysfunction-associated steatotic liver disease (MASLD) is used to harmonize terminology. In studies conducted prior to the nomenclature change, MASLD corresponds to what was previously defined as Nonalcoholic Fatty Liver Disease (NAFLD) according to the diagnostic criteria applied in those original publications. Abbreviations used: AD, Alzheimer’s disease; β, regression coefficient; CI, confidence interval; CT, computed tomography; DSST, Digit Symbol Substitution Test; DTI, diffusion tensor imaging; FA, fractional anisotropy; FHS, Framingham Heart Study; FIB-4, Fibrosis-4 index; FLI, Fatty Liver Index; HR, hazard ratio; kPa, kilopascal; MASLD, metabolic dysfunction-associated steatotic liver disease; MCI, mild cognitive impairment; MD, mean diffusivity; MRI, magnetic resonance imaging; MRI-PDFF, magnetic resonance imaging–proton density fat fraction; PET, positron emission tomography; RS, Rotterdam Study; SHIP, Study of Health in Pomerania; WMH, white matter hyperintensity; WLL, word list learning.

**Table 3:** Blood-based indirect NITs.

Test (Calculation)	Condition	Cutoff	Sensitivity (%)	Specificity/NPV%	Reference
APRI (AST level ÷ ULN ÷ platelet count)	Significant fibrosis due to HBV Cirrhosis due to HCV	>0.35 >1.0	78 76	63 71	Yue et al. [110] Shaheen et al. [111]
FIB-4 (age × AST level) ÷ (platelet count × √ALT level)	Significant fibrosis due to HCV	<1.45 >3.25	60–92 11–54	52–95 91–98	Xu et al. [112]
NFS (−1.675 + (0.037 × age) + (0.094 × BMI) + (1.13 × IR or diabetes [yes = 1, no = 0]) + (0.99 × AST:ALT ratio) − (0.013 × platelet count) − (0.66 × albumin)	Identification of individuals with MASLD at risk of developing fibrosis	−0.835	100	70	Torres et al. [113]

Abbreviations used: ALT, alanine aminotransferase; APRI, aspartate aminotransferase-to-platelet ratio index; AST, aspartate aminotransferase; BMI, body mass index; FIB-4, fibrosis-4 index; HBV, Hepatitis B Virus; HCV, Hepatitis C Virus; IR, insulin resistance; MASLD, metabolic dysfunction–associated steatotic liver disease; NFS, Nonalcoholic Fatty Liver Disease fibrosis score; NPV, negative predictive value; ULN, upper limit of normal. Cirrhosis is defined as METAVIR F = 4; Significant fibrosis is defined as METAVIR F ≥ 2.

**Table 5:** Specific dementia risk algorithms.

Risk Prediction Model	Parameters Included	Comment	References
CAIDE Dementia Risk Score	Age, sex, Education Level, Physical Inactivity, SBP, TChol, BMI.	Originally developed to predict the 20-year dementia risk among middle-aged Finnish individuals, this is the most established and frequently used mid-life risk score for predicting future dementia risk.	Kivipelto et al. [134] Farkas et al. [135]
ANU-ADRI	Age, sex, education level, BMI, diabetes, depression, TChol, traumatic brain injury, smoking, alcohol intake, social engagement, physical activity, cognitive activity, fish intake, and pesticide exposure.	In contrast to constructing risk indices using individual cohort studies, this methodology enables the inclusion of a broader range of risk factors, enhances the generalizability of outcomes, and facilitates the integration of interactions informed by research conducted across various stages of the life course.	Anstey et al. [136]
UKBDRS	Age, education, parental history of dementia, material deprivation, a history of diabetes, stroke, depression, hypertension, high cholesterol, household occupancy, and sex	This is an easy-to-use tool to identify individuals at risk of dementia in the UK. Further research is required to determine the validity of this score in other populations.	Anaturk et al. [137]
CogDrisk tool	Age, sex, education, HTN, midlife obesity, midlife high cholesterol, diabetes, insufficient physical activity, depression, TBI, AF, smoking, social engagement, cognitive engagement, fish consumption, stroke, and insomnia.	A comprehensive risk assessment tool for AD, VaD, and any other type of dementia, which will be applicable in high and low-resource settings.	Anstey et al. [138,139]
LIBRA and LIBRA2	LIBRA focuses on 12 modifiable lifestyle and vascular risk factors, while the updated LIBRA2 version adds three more: hearing impairment, social contact, and sleep.	LIBRA2 demonstrates improved capability in identifying individuals at elevated risk for dementia and serves as an effective tool for public health initiatives focused on reducing dementia risk.	Rosenau et al. [140]

Abbreviations: AD, Alzheimer’s disease; AF, atrial fibrillation; ANU-ADRI, Australian National University’s Alzheimer’s Disease Risk Index; BMI, body mass index; CAIDE, Cardiovascular Risk Factors, Aging, and Dementia; HTN, arterial hypertension; LIBRA, Lifestyle for Brain Health; SBP, systolic blood pressure; TBI, traumatic brain injury; TChol, total cholesterol; UKB-DRP, UK Biobank Dementia Risk Prediction; VaD, vascular dementia.

## Data Availability

No new data were generated.

## Abbreviations

The following abbreviations are used in this manuscript:

β	Regression coefficient
AD	Alzheimer’s disease
AF	Atrial fibrillation
ALD	Alcohol-related liver disease
ALT	Alanine aminotransferase
APOE	Apolipoprotein E
APRI	Aminotransferase–platelet ratio index
AST	Aspartate aminotransferase
AST/ALT	Aspartate aminotransferase/alanine aminotransferase ratio
BBB	Blood–brain barrier
CI	Confidence interval
CLD	Chronic liver disease
DBil	Direct bilirubin
ECM	Extracellular matrix
FHS	Framingham Heart Study
ELF	enhanced liver fibrosis score
FA	Fractional anisotropy
FIB-4	Fibrosis-4 index
GGT	Gamma-glutamyl transpeptidase
GLP-1 RAs	Glucagon-like peptide-1 receptor agonists
HOMA	Homeostatic model assessment
HR	Hazard ratio
HSC	Hepatic stellate cell
MASH	Metabolic dysfunction-associated steatohepatitis
MASLD	Metabolic dysfunction-associated steatotic liver disease
MD	Mean diffusivity
MetALD	Metabolic dysfunction and ALD
MMSE	Mini-Mental State Examination
MoCA	Montreal Cognitive Assessment
MRE	Magnetic Resonance Elastography
MRI	Magnetic resonance imaging
MRI-PDFF	Magnetic resonance imaging–proton density fat fraction
NFS	NAFLD fibrosis score
PDD	Parkinson’s Disease Dementia
PET	Positron emission tomography
PFDR	False discovery rate-adjusted *p*-value
PNPLA3	Patatin-like phospholipase domain-containing protein 3
RS	Rotterdam Study
SHIP	Study of Health in Pomerania
TBil	Total bilirubin
T2D	Type 2 diabetes
TMAO	Trimethylamine-N-oxide
UK	United Kingdom
VaD	Vascular dementia
VCTE	Vibration-controlled transient elastography
WMH	White matter hyperintensity

## Appendix A

**Table A1:** Search syntax used to identify studies evaluating the association between liver fibrosis and dementia.

Database	Syntax
PubMed	((“liver”[Mesh] AND (“Elasticity Imaging Techniques”[Mesh] *OR* “biopsy”[Mesh] *OR* “fibrosis”[Mesh])) *OR* “Liver Cirrhosis”[Mesh] *OR* ((“liver”[tiab] *OR* “hepatic”[tiab]) AND (“biops*”[tiab] *OR* “fibros*”[tiab] *OR* “cirrhos*”[tiab] *OR* “stiffness*”[tiab] *OR* “puncture”[tiab] *OR* “elastogra*”[tiab] *OR* “elasticit*”[tiab] *OR* “acoustography”[tiab] *OR* “vibroacoustography”[tiab] *OR* “vibro-acoustography”[tiab] *OR* “sonoelastograph*”[tiab] *OR* “fibroscan”[tiab] *OR* “acoustic radiation force impulse imaging”[tiab] *OR* “arfi imaging*”[tiab]))) *AND* (“Dementia”[Mesh] *OR* “Dementia*”[tiab] *OR* “Alzheimer*”[tiab] *OR* “Binswanger encephalopathy”[tiab] *OR* “CADASIL”[tiab] *OR* “Lewy body disease”[tiab] *OR* “Neurofibrillary tangles with calcification”[tiab] *OR* “Primary progressive aphasia”[tiab] *OR* “Progressive nonfluent aphasia”[tiab] *OR* “Hereditary diffuse leukoencephalopathy with spheroids”[tiab] *OR* “Huntington chorea”[tiab] *OR* “Kluver-Bucy syndrome”[tiab] *OR* “Mental deterioration”[tiab] *OR* “Nasu-Hakola disease”[tiab] *OR* “Neuronal ceroid lipofuscinosis”[tiab] *OR* “Prion disease”[tiab] *OR* “Bovine spongiform encephalopathy”[tiab] *OR* “Chronic wasting disease”[tiab] *OR* “Creutzfeldt-Jakob disease”[tiab] *OR* “Feline spongiform encephalopathy”[tiab] *OR* “Fatal familial insomnia”[tiab] *OR* “Gerstmann-Straussler-Scheinker syndrome”[tiab] *OR* “Kuru”[tiab] *OR* “Scrapie”[tiab] *OR* “Transmissible mink encephalopathy”[tiab] *OR* “Variably protease-sensitive prionopathy”[tiab] *OR* “Pseudodementia”[tiab] *OR* “Rett syndrome”[tiab] *OR* “Senility”[tiab] *OR* “Tauopathy”[tiab] *OR* “Creutzfeldt-Jakob syndrome”[tiab] *OR* “Diffuse neurofibrillary tangles with calcification”[tiab] *OR* “Frontotemporal lobar degeneration”[tiab] *OR* “Huntington disease”[tiab] *OR* “Amentia*”[tiab]) *AND* (“1900/01/01”[Date-Publication]: “2025/11/30”[Date-Publication]) *AND* (humans[Filter]) *AND* (english[Filter]) *AND* (alladult[Filter])

**Note:** MeSH = Medical Subject Headings; [tiab] = search in title/abstract; [Date-Publication] = publication date filter; [Filter] = population/language filter. Boolean operators: AND = include both terms; OR = include any term. Parentheses () = group terms; * (asterisk) = wildcard to capture multiple word endings. The search was limited to studies on humans, those in English, and those conducted on adults, from inception through to 30 November 2025.
